# Serum calprotectin—a promising diagnostic marker for adult-onset Still’s disease

**DOI:** 10.1007/s10067-015-3108-6

**Published:** 2015-11-07

**Authors:** Qian Guo, Xicao Zha, Chun Li, Yuan Jia, Lei Zhu, Jianping Guo, Yin Su

**Affiliations:** Department of Rheumatology and Immunology, Peking University People’s Hospital, 11 South Xizhimen Street, Beijing, 100044 China; Department of Traditional Chinese Medicine, North Branch of People’s Hospital of Xinjiang Uygur Autonomous Region, Urumqi, 830054 China

**Keywords:** Adult-onset Still’s disease, Calprotectin, Diagnostic marker

## Abstract

Calprotectin is a calcium-binding cytosolic protein, mainly expressed in immune cells, such as neutrophils, monocytes, and macrophages. Our study aimed to evaluate the diagnostic value of calprotectin for adult-onset Still’s disease (AOSD), by comparing serum calprotectin concentrations in patients with AOSD (*n* = 46), rheumatoid arthritis (RA, *n* = 34), primary Sjögren syndrome (pSS, *n* = 40), systemic lupus erythematosus (SLE, *n* = 39), osteoarthritis (OA, *n* = 20), and healthy controls (HCs, *n* = 49). Calprotectin concentrations were significantly higher in patients with AOSD (55.26 ± 18.00 ng/ml), compared to patients with RA (39.17 ± 18.90 ng/ml), pSS (35.31 ± 19.47 ng/ml), SLE (32.21 ± 25.01 ng/ml), OA (19.24 ± 10.67 ng/ml), and HCs (8.46 ± 5.17 ng/ml). All the differences were highly significant (*p* < 0.001). Using receiver-operating characteristic curve, the cut-off value of calprotectin was defined as 45.488 ng/ml, and its sensitivity and specificity for AOSD diagnosis were 63.0 and 80.1 %, respectively. The positive rate of calprotectin was significantly higher in AOSD cases compared to patients with other diseases and healthy controls (*p* < 0.001). Serum calprotectin was positively correlated with ferritin (*r* = 0.294, *p* < 0.05), and concentration of hemoglobin was significantly lower in calprotectin-positive patients compared to negative patients in AOSD (103.49 ± 20.21 g/l vs 115.71 ± 15.59 g/l, *t* = −2.142, *p* = 0.038). These findings suggest that serum calprotectin may serve as a promising marker for the diagnosis of AOSD and monitor disease activity to a certain extent.

## Introduction

Adult-onset Still’s disease (AOSD) is a systemic disease with unclear etiology, characterized by spiking fever, arthritis, evanescent rash, lymphadenopathy, hepato-splenomegaly, sore throat, neutrophilic leukocytosis, and abnormality of liver enzymes [1]. Before the diagnosis of AOSD, infectious, neoplastic, and other rheumatological disorders should be ruled out. AOSD is self-limiting, but more than one third of patients develop severe systematic damage with a poor prognosis [1, 2]. Since there were no disease-specific manifestations and serologic markers, the diagnosis for AOSD and accurate determination of disease activity is difficult [3, 4]. The currently used markers for the diagnosis of AOSD included erythrocyte sedimentation rate (ESR), C-reactive protein (CRP), and serum ferritin, which lacking the sensitivity, specificity, and precision to diagnose AOSD and to monitor the disease activity. Thus, specific serological marker for diagnosis and assessment of disease activity in AOSD is urgently needed.

Calprotectin is a calcium-binding cytosolic protein that belongs to the S100 family, which presents in regenerative cells such as neutrophils, monocytes, macrophages, epithelial, and endothelial cells [5]. It is widely distributed in human tissues, body fluids, and cells. In neutrophils, calprotectin distributes in the cytosol outside lysosomes and constitutes approximately 5 % of the total cellular proteins and is a marker for neutrophil’s update [6]. Calprotectin may involve modulation of inflammatory and calcium-dependent regulation of protein phosphorylation, transcription, and enzyme activities [7, 8]. Furthermore, previous studies have showed that calprotectin was related to disease activity in several inflammatory diseases, such as juvenile rheumatoid arthritis (JRA) [9–11], reactive arthritis [12], acute gouty arthritis [13], and SLE [14]. In patients with RA, calprotectin was detected in the inflamed synovial and has increased in plasma and synovial fluid from affected joints, which proves it is strongly correlated with joint inflammation and damage in RA [15]. Likewise, the concentrations of calprotectin were also elevated in intestinal mucosa, serum, and feces in patients with Crohn’s disease and ulcerative colitis [16, 17]. One publication reported that the expression levels of serum calprotectin were elevated in patients with AOSD and correlated with disease activity and severity but with the comparison between AOSD cases and healthy controls only [18].

Our study aimed to evaluate the diagnostic value of calprotectin for AOSD, by comparing the serum levels of calprotectin in AOSD patients with several other autoimmune disease controls, i.e., rheumatoid arthritis (RA), primary Sjögren syndrome (pSS), systemic lupus erythematosus (SLE), osteoarthritis (OA), as well as with healthy controls (HCs). The correlation between calprotectin and disease activity/severity was also analyzed at the same time. Our results indicate that calprotectin could be a promising marker for AOSD diagnosis and monitor disease activity to a certain extent.

## Materials and methods

### Subjects

Serum samples were collected from 46 patients with AOSD, 34 patients with RA, 40 patients with pSS, 39 patients with SLE, 29 patients with OA, and 49 HCs. The AOSD patients fulfilled the criteria for a diagnosis of AOSD, with the exclusion of patients who had infections, hematological, and other autoimmune diseases [19]. Patients suffering from other autoimmune diseases were selected according to the disease-specific diagnostic criteria [20–23]. Patients with either disease were selected without development of other rheumatic diseases. All the patients were recruited from the Department of Rheumatology and Immunology at Peking University People’s Hospital. The patients were not subject to medical treatment at the time of serum samples obtained. The 49 non-related healthy controls were recruited from Health Care Centers from Peking University People’s Hospital and were selected without any disease records.

All sera were kept at −20 °C. The baseline demographic characteristics of patients and controls are shown in Table 1. This study was approved by the ethics committee of Peking University People’s Hospital, and informed consent was received from all study subjects.

**Table 1:** General characteristics of patients and controls. All values presented as number or mean ± SD

	AOSD	RA	pSS	SLE	OA	HC
N	46	34	40	39	29	49
Age (years, mean ± SD)	36.6 ± 17.5	56.9 ± 9.9	55.4 ± 12.2	40.0 ± 15.4	56.6 ± 7.1	35.6 ± 9.7
Female/male	23/23	27/7	34/6	29/10	25/4	23/26
Disease duration (years, mean ± SD)	1.3 ± 1.8	8.6 ± 6.9	11.4 ± 10.3	6.0 ± 5.9	7.8 ± 6.9	

### Clinical and laboratory assessment

Clinical features of AOSD patients, including fever, evanescent rash, sore throat, arthritis, serositis, hepatomegaly, splenomegaly, and lymphadenopathy were recorded. Complete blood cell count, erythrocyte sedimentation rate (ESR), C-reactive protein (CRP), serum ferritin, glutamic pyruvic transaminase (ALT), and glutamic oxalacetic transaminase (AST) and serum levels of IgG, IgA, and IgM were detected.

### Measurement of calprotectin concentrations

Serum concentrations of calprotectin were measured using commercial ELISA kits (Sun Biomedical Technology Co., Ltd, Beijing, China) according to the manufacturer’s protocol. Each 100 μl sample of serum or standard was placed in a microtiter plate well coated with a monoclonal antibody to human calprotectin heterodimer. After incubation for 1 h at room temperature, the wells were washed four times with buffer, and samples were labeled with 100 μl of dilute tracer (biotinylated secondary antibody to human calprotectin). After washing again, each well received 100 μl of diluted streptavidin-peroxidase conjugate, which reacts specifically with the calprotectin-bound tracer. After incubation at room temperature for 1 h, excess conjugate was removed by washing, and tetramethylbenzidine substrate solution (100 μl) was applied to each well for 30 min in the dark at room temperature. To terminate the reaction, stop solution containing citric acid (100 μl) was added to each well, and absorbance was measured at 450 nm using an ELISA plate reader (Bio-Rad, CA, USA). The coefficient of variation was <6 % for duplicate measurements by ELISA.

### Statistical analysis

The Statistical Package for Social Sciences version 16.0 (SPSS, Chicago, IL, USA) was used to analyze the data. Results are shown as mean ± SD. The results were analyzed by independent *t* tests, Mann–Whitney *U* test, and chi-square tests as appropriate. The ability of calprotectin to diagnose AOSD was evaluated by a receiver-operating characteristics (ROC) curve analysis. The correlations between serum calprotectin level and disease-related variables were evaluated with Pearson or Spearman’s correlation test. A *p* value <0.05 was regarded as statistically significant.

## Results

### Clinical characteristics of the AOSD patients

In AOSD patients, the main clinical symptoms include fever (100 %), evanescent rash (73.9 %), arthritis (50 %), sore throat (71.7 %), serositis (26.1 %), hepatomegaly (28.2 %), splenomegaly (50 %), and lymphadenopathy (50 %). The general and clinical characteristics are summarized in Tables 1 and 2, respectively.

**Table 2:** Clinical characteristics of AOSD patients

Clinical manifestations	*N* (%)	Annotation
Fever	46 (100)	In the course of disease, the temperature in all patients were fluctuate from 35.95 ± 0.34 °C to 39.74 ± 0.61 °C
Evanescent rash	34 (73.9)	Often appear with fever, the rash is transient, dissipate with defervescence
Sore throat	33 (71.7)	Patients’ complaint
Arthritis	23 (50)	Mainly showed by joint tenderness and pain, swelling is rare
Serositis	12 (26.1)	Demonstrated by radiography
Hepatomegaly	13 (28.2)	Demonstrated by radiography
Splenomegaly	23 (50)	Demonstrated by radiography
Lymphadenopathy	23 (50)	Demonstrated by radiography and physical examination

### Sensitivity and specificity of calprotectin in diagnosis of AOSD

**Fig. 1:**
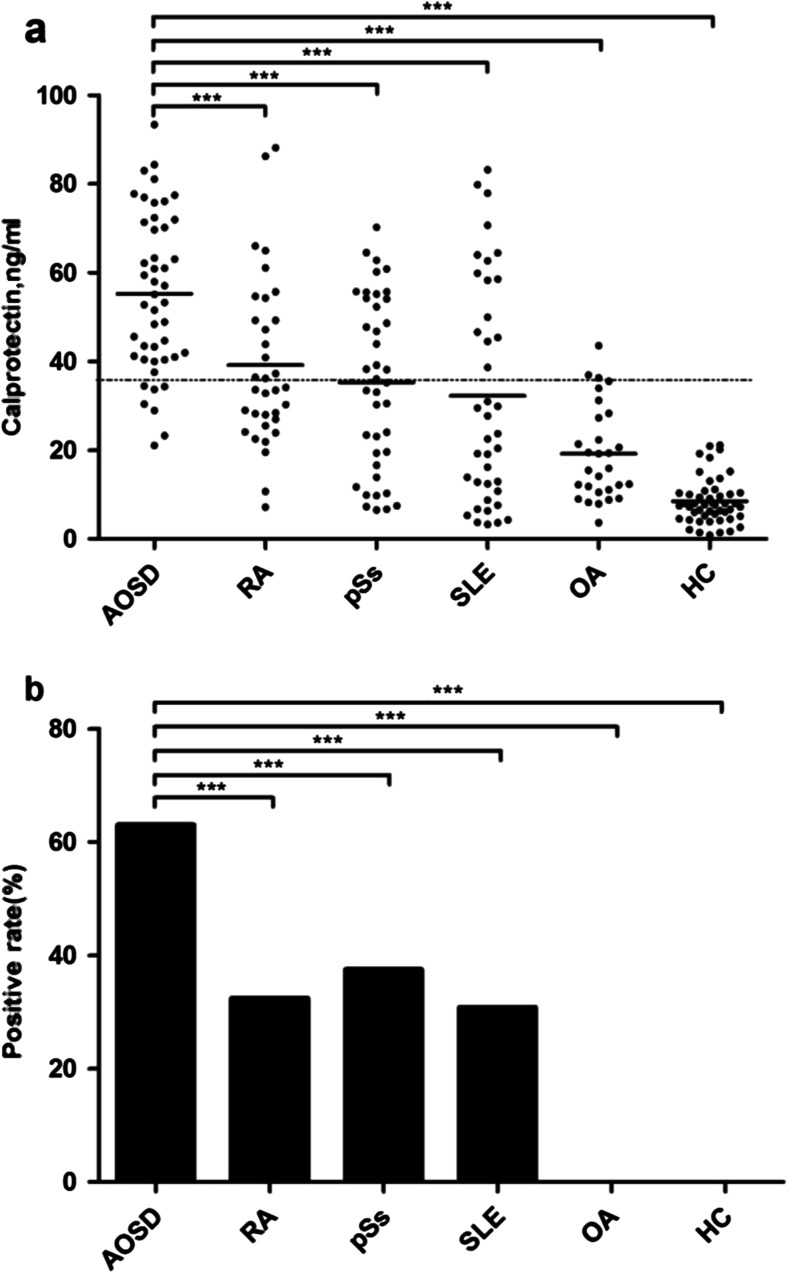
**a** Calprotectin levels in AOSD patients and controls. *Each dot* represents an individual serum. *Bars* display median and *dotted line* represents cut-off values 45.488. Mean calprotectin levels were significantly higher in patients with AOSD (55.26 ± 18.0 ng/ml), RA (39.17 ± 18.90 ng/ml), pSS (35.31 ± 19.47 ng/ml), SLE (32.21 ± 25.01 ng/ml), OA (19.24 ± 10.67 ng/ml) than in HC (8.46 ± 5.17 ng/ml) (*p* < 0.001). Calprotectin levels were significantly higher in patients with AOSD than in patients with RA, pSS, SLE, OA, and HC (*p* < 0.001). **b** The positive rate of calprotectin in patients with AOSD and controls. The positive rate of calprotectin in patients with AOSD (63.0 %, 29/46) were significantly higher than those in patients with RA (32.4 %, 11/34), pSS (37.5 %, 15/40), SLE (30.8 %, 12/39), OA (0 %, 0/29), HC (0 %, 0/49) (*p* < 0.001). ***p < 0.001

As shown in Fig. 1a, mean levels of calprotectin were significantly higher in patients with AOSD (55.26 ± 18.0 ng/ml), RA (39.17 ± 18.90 ng/ml), pSS (35.31 ± 19.47 ng/ml), SLE (32.21 ± 25.01 ng/ml), OA (19.24 ± 10.67 ng/ml), compared to HCs (8.46 ± 5.17 ng/ml); all the differences were statistically significant (*p* < 0.001). Furthermore, calprotectin levels were significantly higher in patients with AOSD than in patients with other autoimmune diseases those mentioned above (*p* < 0.001, Fig. 1a). Using ROC curve, the calculated cut-off value of calprotectin was 45.488 ng/ml. With the cut-off value, the positive rate of calprotectin in patients with AOSD (29/46, 63.0 %) was significantly higher than those in patients with other rheumatic diseases, including RA (11/34, 32.4 %), pSS (15/40, 37.5 %), SLE (12/39, 30.8 %), OA (0/29, 0 %), as well as HCs (0/49, 0 %) (*p* < 0.001, Fig. 1b). The sensitivity and specificity of serum calprotectin were 63.0 and 80.1 % for AOSD, respectively. The positive and negative predictive values of the calprotectin in AOSD were 43.3 and 90.0 %. Thus, the calprotectin would be considered to be “good” or “fair” at separating AOSD patients from individuals with other rheumatic diseases and health controls.

### Associations of serum calprotectin levels with serologic parameters in AOSD

Next, we evaluated whether a positive test of serum calprotectin could be correlated to other serologic parameters in AOSD. As shown in Table 3, the levels of serum calprotectin were significantly correlated with serum ferritin in AOSD (*r* = 0.294, *p* = 0.048). The level of hemoglobin (Hb) was significantly lower in serum calprotectin-positive patients (103.49 ± 20.21 g/l), compared to serum calprotectin negative patients (115.71 ± 15.59 g/l), (*t* = −2.142, *p* = 0.038). The concentration of ESR and CRP were elevated in calprotectin-positive patients, as compared to negative patients, (ESR: 79.14 ± 34.98 mm/h vs 60.24 ± 33.23 mm/h, *p* = 0.077. CRP: 100.78 ± 58.90 mg/l vs 68.78 ± 56.02 mg/l, *p* = 0.076), though being unable to reach the statistical significance. There was no significant difference in white blood cell (WBC), blood platelet (PLT), ALT, AST, IgG, IgA, and IgM between the two groups (*p* > 0.05) (Table 4).

**Table 3:** The correlation of calprotectin and disease-related laboratory data in AOSD

Laboratory data	*r*	*p* values
Ferritin	0.294	0.048
ESR	0.242	0.105
CRP	0.093	0.537
WBC	0.269	0.071
Hb	−0.267	0.073
PLT	0.136	0.368
ALT	−0.024	0.875
AST	−0.009	0.951
IgG	−0.054	0.722
IgA	−0.038	0.802
IgM	−0.012	0.938

*ESR* erythrocyte sedimentation rate, *CRP* C-reactive protein, *WBC* white blood cell, *Hb* hemoglobin, *PLT* platelet, *ALT* glutamic pyruvic transaminase, *AST* glutamic oxalacetic transaminase

**Table 4:** Divided AOSD patients into calprotectin-positive and calprotectin-negative groups by the calprotectin cut-off value, the comparison of laboratory data between the two groups

Laboratory data	Calprotectin-positive (*n* = 29)	Calprotectin-negative (*n* = 17)	*p* values
Ferritin (ng/ml)	1536.20 ± 647.53	1300.80 ± 686.18	0.250
ESR (mm/h)	79.14 ± 34.98	60.24 ± 33.23	0.077
CRP (mg/l)	100.78 ± 58.90	68.78 ± 56.02	0.076
WBC (10^9/l)	17.89 ± 4.69	16.24 ± 5.40	0.284
Hb (g/l)	103.49 ± 20.21	115.71 ± 15.59	0.038
PLT (10^9/l)	336.91 ± 162.68	334.24 ± 63.77	0.949
ALT (U/l)	106.48 ± 169.69	141.29 ± 194.95	0.528
AST (U/l)	93.83 ± 163.84	76.06 ± 118.58	0.698
IgG (g/l)	12.02 ± 5.61	13.05 ± 2.84	0.484
IgA (g/l)	4.18 ± 4.54	3.07 ± 1.28	0.224
IgM (g/l)	1.50 ± 0.82	1.36 ± 0.79	0.563

*ESR* erythrocyte sedimentation rate, *CRP* C-reactive protein, *WBC* white blood cell, *Hb* hemoglobin, *PLT* platelet, *ALT* glutamic pyruvic transaminase, *AST* glutamic oxalacetic transaminase

### Correlation between serum calprotectin levels and clinical manifestations in AOSD

Serum calprotectin in AOSD patients showed no correlation with their age and gender (*p* > 0.05). Except the proportion of patients with sore throat was higher in calprotectin negative group than the positive group (*p* = 0.017), there was no significant difference between the two groups in other clinical features including fever, evanescent rash, arthritis, serositis, hepatomegaly, splenomegaly, and lymphadenopathy (*p* > 0.05, Table 5).

**Table 5:** The correlation of calprotectin and clinical manifestations in AOSD

Clinical features	Calprotectin-positive (*n* = 29)		Calprotectin-negative (*n* = 17)		*χ* ^2^	*p* values
	*n*	%	*n*	%		
Fever	29	100	17	100	3.695	0.055
Evanescent rash	21	72.4	13	76.5	0	1
Sore throat	18	62.1	15	88.2	5.698	0.017
Arthritis	14	48.3	9	52.9	0.093	0.760
Serositis	10	34.5	2	11.8	1.811	0.178
Hepatomegaly	10	34.5	3	17.6	0.783	0.376
Splenomegaly	16	55.2	7	41.2	2.339	0.126
Lymphadenopathy	15	51.7	8	47.1	0.093	0.760

## Discussion

AOSD is a systemic disease of unknown etiology. Although it is self-limiting, more than one third of patients develop severe systematic damage with a poor prognosis [2]. The diagnostic spectrum of AOSD is wide but lacking the disease-specific manifestations and serologic markers for accurate diagnosis of AOSD and assess of disease activity and severity [3, 4]. Former studies have shown follistatin-like protein 1 could be a useful biomarker for systemic JIA activity [24], but it failed to present its use for disease activity in AOSD [25]. Although previous studies have proposed IL-18 [26, 27], serum soluble intracellular adhesion molecule-1 (sICAM-1) [2], and macrophage migration inhibitory factor (MIF) [28] for AOSD activity and/or severity, there were many limitations for these markers to put into clinical utility. Serum ferritin is the most frequently used laboratory index for AOSD diagnosis and activity [1], but it can also increase during a non-specific inflammation and may be affected by iron concentration in the body. ESR and CRP are also used for evaluation of AOSD activity, but they are so common in inflammation or no-inflammation diseases, thus lacking specificity for AOSD. Calprotectin which is supposed to be involved in the recruitment of inflammatory cells to sites has been deemed to form “damage-associated molecular patterns” of injury [29, 30]. It has been confirmed to exert an influence in several inflammatory diseases such as JRA, reactive arthritis, acute gouty arthritis, and SLE [9–14]. In patients with systemic-onset JRA, calprotectin was highly expressed in patients with active disease and made them good candidates as markers for the diagnosis of systemic-onset JIA and monitoring disease activity [10, 11]. In patients with RA, calprotectin has been proved to possess strong correlation with joint inflammation and damage [15]. Results from 42 patients with polymyalgia rheumatica or temporal arteritis showed that serum calprotectin levels are highly correlated with the acute phase clinical manifestations of the disease and its related parameters such as ESR, CRP, etc., and calprotectin levels were significantly decreased after the treatment with glucocorticoids [31]. These findings suggest that serum calprotectin levels can provide reliable clinical indicators for monitoring disease activity and severity of rheumatic diseases.

Although one study has shown that serum calprotectin increased in patients with AOSD and was closely related with disease activity and severity, the work merely compared the serum calprotectin concentrations between AOSD patients and healthy individuals [18]. To better evaluate the value of calprotectin for AOSD diagnosis, in present work, we not only included healthy individuals as controls but also utilized several disease controls to discriminate AOSD from other rheumatic diseases. Our data indicate that serum calprotectin levels were significantly elevated in patients with AOSD, RA, pSS, SLE, and OA than HCs. Furthermore, calprotectin levels were significantly higher in patients with AOSD than in patients with other rheumatic diseases mentioned above. These data may help us to distinguish AOSD from a number of rheumatic diseases with frequent manifestation of arthritis.

Using ROC curve, the cut-off value of calprotectin was defined as 45.488 ng/ml, with a sensitivity and specificity being 63.0 and 80.1 %, respectively. Serum calprotectin in patients with AOSD showed no obvious relevance to their age and gender as well as the patient’s fever, evanescent rash, arthritis, serositis, hepatomegaly, splenomegaly, and lymphadenopathy. However, our data showed that the proportion of patients with sore throat was higher in calprotectin negative group than the positive group, which has not been reported before. In a review of 341 AOSD cases, the incidence of sore throat was reported to be 69 % and was particularly displayed prevalent in the first month of disease course [32]. In our study, the incidence of sore throat in AOSD is 71.7 %, which is consistent with previous studies. At present, we are unable to give a clear explanation for the higher incidence of sore throat in calprotectin negative patients, but we assume that it could be duo to the appearance of sore throat and the increase of calprotectin occurred at different stages of the diseases. Further functional studies will hopefully elucidate the role of calprotectin involved in the manifestation in the future. Regarding serologic parameters, serum calprotectin had strong correlation with serum ferritin in AOSD patients, which has been reported previously [18]. Interestingly, our data indicated that serum calprotectin was negatively correlated with Hb in AOSD patients. The patients who were positive for calprotectin had lower Hb content. This funding agrees with the characteristics of anemia in chronic inflammatory disease and the anemia in AOSD has also been reported in previous studies [4]. The anemia condition can be remitted during the disease’s asymptomatic periods and can also return during its exacerbations [33]. Meanwhile, our data showed that the levels of ESR and CRP were higher in serum calprotectin-positive patients than in negatives, suggesting that calprotectin together with these two inflammatory markers may provide messages for disease activity. Previously, Jung SY et al. reported that serum calprotectin has strong correlations with WBC, CRP, AST, but not with ESR, Hb, or PLT [18]. In our study, there were no associations between calprotectin and WBC, PLT, ALT/AST, IgG, IgA, and IgM. This may be explained by the divergence of the study case numbers between ours (*n* = 46) and Jung’s work (*n* = 25).

In conclusion, serum calprotectin levels were significantly increased in AOSD and it is correlated with serum ferritin and Hb. It can be a promising marker for AOSD diagnosis and monitoring disease activity to a certain extent. The limitation of this study is the modest sample size. A prospective study with larger numbers of cases and a close follow-up of patients are needed to reveal the expression changes of calprotectin that accompany disease activation, remission, and treatment more precisely.
